# An immune subtype-related prognostic signature of hepatocellular carcinoma based on single-cell sequencing analysis

**DOI:** 10.18632/aging.204012

**Published:** 2022-04-12

**Authors:** Jiaheng Xie, Liang Chen, Qingmei Sun, Haobo Li, Wei Wei, Dan Wu, Yiming Hu, Zhechen Zhu, Jingping Shi, Ming Wang

**Affiliations:** 1 Department of Burn and Plastic Surgery, The First Affiliated Hospital of Nanjing Medical University, Nanjing, Jiangsu, China; 2 Department of General Surgery, Fuyang Hospital Affiliated to Anhui Medical University, Fuyang, Anhui, China; 3 Pancreas Center, The First Affiliated Hospital with Nanjing Medical University, Nanjing, Jiangsu, China; 4 Graduate School of Peking Union Medical College, Chinese Academy of Medical Sciences, Beijing, Beijing, China; 5 Department of Rheumatology and Immunology, Nanjing Drum Tower Hospital, The Affiliated Hospital of Nanjing University Medical School, Nanjing, Jiangsu, China; 6 College of Pharmacy, Jiangsu Ocean University, Lianyungang, Jiangsu, China

**Keywords:** hepatocellular carcinoma, single-cell sequencing analysis, differential expression analysis, immune microenvironment, prognostic model

## Abstract

Hepatocellular carcinoma (HCC) is one of the most common cancers in the world and is often associated with a poor prognosis. The main reason for this poor prognosis is that inconspicuous early symptoms lead to delayed diagnosis. Treatment options for advanced HCC remain limited and ineffective. In this context, the exploration of the immune microenvironment in HCC becomes attractive. In this study, we divided HCC into immune cell and non-immune cell subtypes, by single-cell sequencing analysis of GEO dataset GSE146115. We found differentially expressed genes in the two subtypes, which we used to construct a prognostic model for HCC through Cox and Lasso regressions. Our prognostic model can accurately evaluate the prognosis of HCC patients, and provide a reference for the design of immunotherapy for HCC.

## INTRODUCTION

Hepatocellular carcinoma (HCC) is the most common type of primary liver cancer and a serious threat to human health, hindering social and economic development [1, 2]. Currently, HCC is the fifth most common type of cancer worldwide [3]. HCC has a high mortality rate and is the second leading cause of cancer death [4]. Early-stage HCC can be treated by radiofrequency ablation, trans-arterial therapy, and surgical resection [5]. Unfortunately, a large proportion of HCC patients are diagnosed with advanced-stage disease because the symptoms of early-stage HCC are not obvious [6]. Treatment for advanced HCC is often limited [7]. In recent years, targeted therapy and immunotherapy have achieved good initial results in the treatment of HCC [8]. Targeting therapy with sorafenib for HCC and immunotherapy with immune checkpoint inhibitors such as nivolumab are both promising treatment options [9]. It is worth mentioning that the immune microenvironment of HCC plays an indelible role in its onset, progression, and response to treatment [10]. It is time to study the immune microenvironment of HCC further.

As the frontier of genomics, single-cell sequencing technology is developing rapidly [11]. This allowed us to precisely study the immune landscape of multiple cells in HCC. Moreover, it provides a way to cluster and annotate cells to better understand tumour immune mechanisms, the emergence of drug resistance, and changes in cell differentiation in HCC [12]. Single-cell sequencing technology will bring exciting breakthroughs in tumour genomics in the future.

In this study, we first used single-cell sequencing analysis to perform dimension reduction, clustering, and cell annotation for HCC. This is where we were able to divide the different cells from HCC into immune and non-immune groups. We then investigated the differential gene expression between the two immune subsets and constructed a prognostic model of HCC based on these genes. This prognostic model can accurately assess the prognosis of HCC patients and is related to the immune microenvironment and drug sensitivity. Our study can provide a new idea for the diagnosis and treatment of HCC.

## RESULTS

### Quality control of single-cell sequencing data

**Figure 1:**
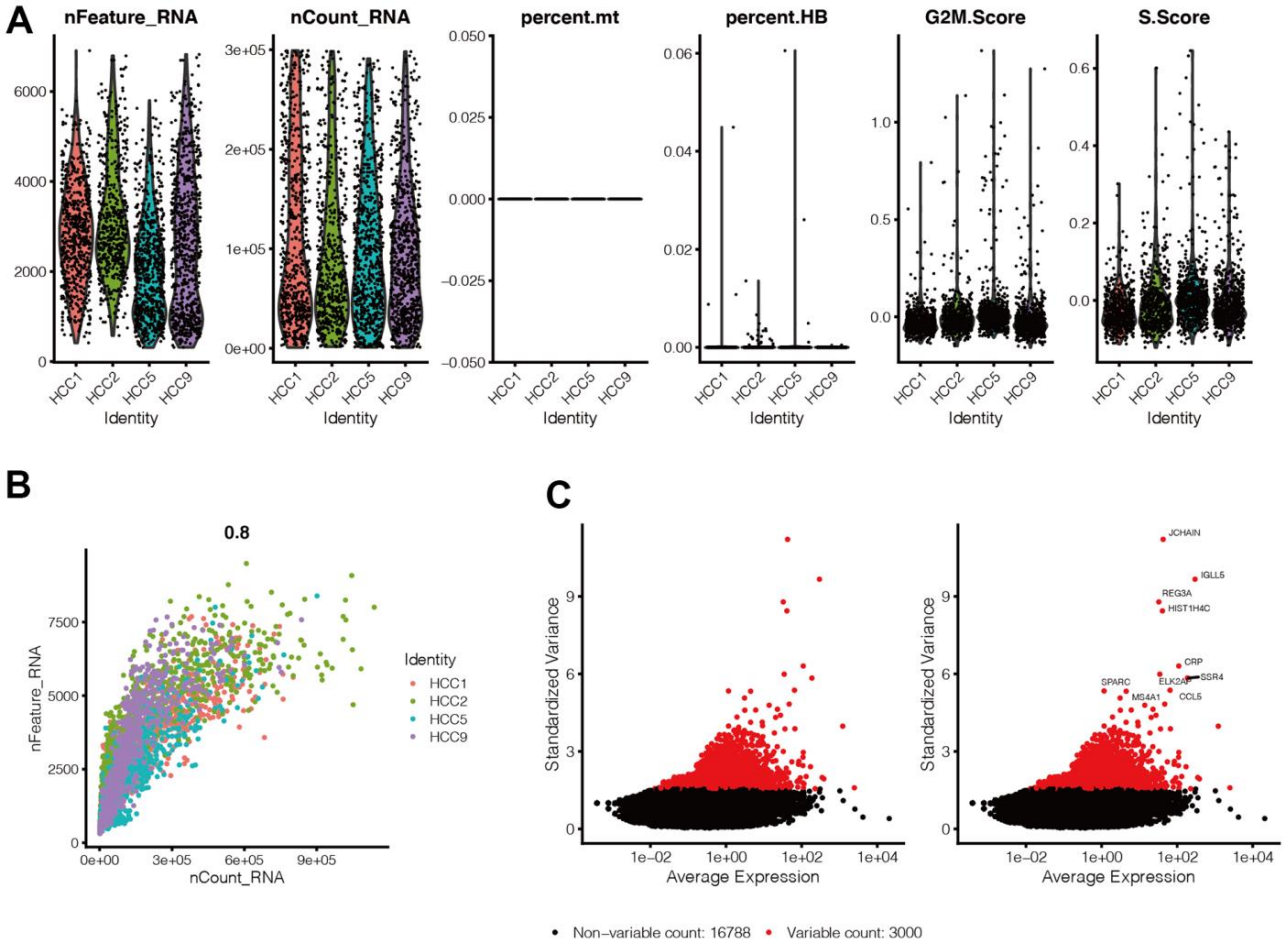
**Quality control of single-cell sequencing data.** (**A**) Gene expression levels in each cell of the 4 samples were in the range of 300-7000, and the distribution was relatively uniform. At the same time, we found that the percentage of mitochondrial genes was almost 0, and the percentage of red blood cell genes was less than 1, and the scores of G2M phase and S phase of the cell cycle were evenly distributed in the four samples. (**B**) Cells are evenly distributed in the four samples, and the number of genes is positively correlated with the expression level of genes, with a correlation of 0.8. (**C**) We selected 300 hypervariable genes from all the genes, which are in red, and the first 10 genes were flagged.

By setting the criteria for screening the cells, we ended up with 2,587 cells. As shown in Figure 1A, we found that gene expression levels in each cell of the four samples were in the range of 300-7,000, and the distribution was relatively uniform. At the same time, we also found that mitochondrial genes were almost 0%, and red blood cell genes were <1%, with the scores of G2M and S phases of the cell cycle evenly distributed in the four samples. Figure 1B shows that cells are evenly distributed in the four samples, and the number of genes is positively correlated with their expression level (0.8). The above results suggest that the cells obtained by filtering can be used for subsequent analysis. In Figure 1C, we selected 300 hypervariable genes, which are in red, and the first ten genes were flagged.

### Acquisition of characteristic genes associated with immune subtypes

**Figure 2:**
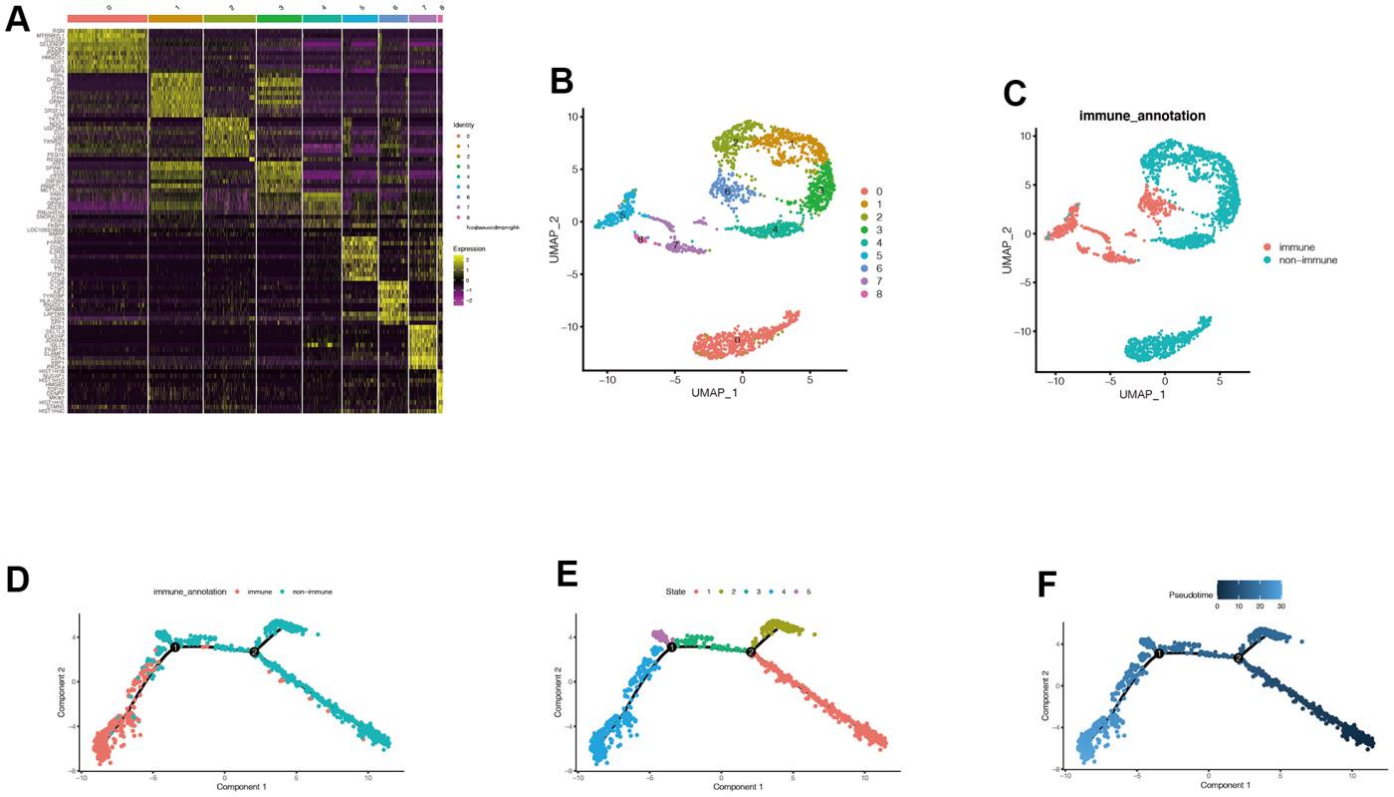
**Acquisition of characteristic genes associated with immune subtypes.** (**A**) After dimension reduction through PCA, we found that the cells were clustered into 9 clusters. It can be seen that the expression of the first 10 genes in each cluster is significantly higher than that in other clusters. (**B**, **C**) We can find the distribution of these 9 clusters, and the cluster labels corresponding to immune cells are 5,6,7, and 8. Cells can be divided into immune cells and non-immune cells. (**D**–**F**) We then explored the differentiation trajectory of immune and non-immune cells. With the time change of cell differentiation, that is, cells differentiate from the deeper blue branches to the lighter blue branches, the differentiation states of immune cells and non-immune cells are different, and the differentiation time of non-immune cells may be earlier. Then we obtained 526 genes related to immune subtypes by using the Findmarkers function.

After dimension reduction through PCA, we found that the cells were organized in nine clusters. Figure 2A showed the distribution of the first ten differential genes in each cluster. The expression of the first ten genes in each cluster is significantly higher than in other clusters. In Figure 2B, 2C, we can find the distribution of these nine clusters, and the cluster labels corresponding to immune cells are 5, 6, 7, and 8. We then explored the differentiation trajectory of immune and non-immune cells. Figure 2D–2F shows the difference in timing of cell differentiation. As cells differentiate from the deeper blue branches to the lighter blue branches, non-immune cells differentiate earlier than immune cells. Then, we obtained 526 genes related to immune subtypes by using the Findmarkers function.

### Enrichment analysis of immune subtypes-related genes

**Figure 3:**
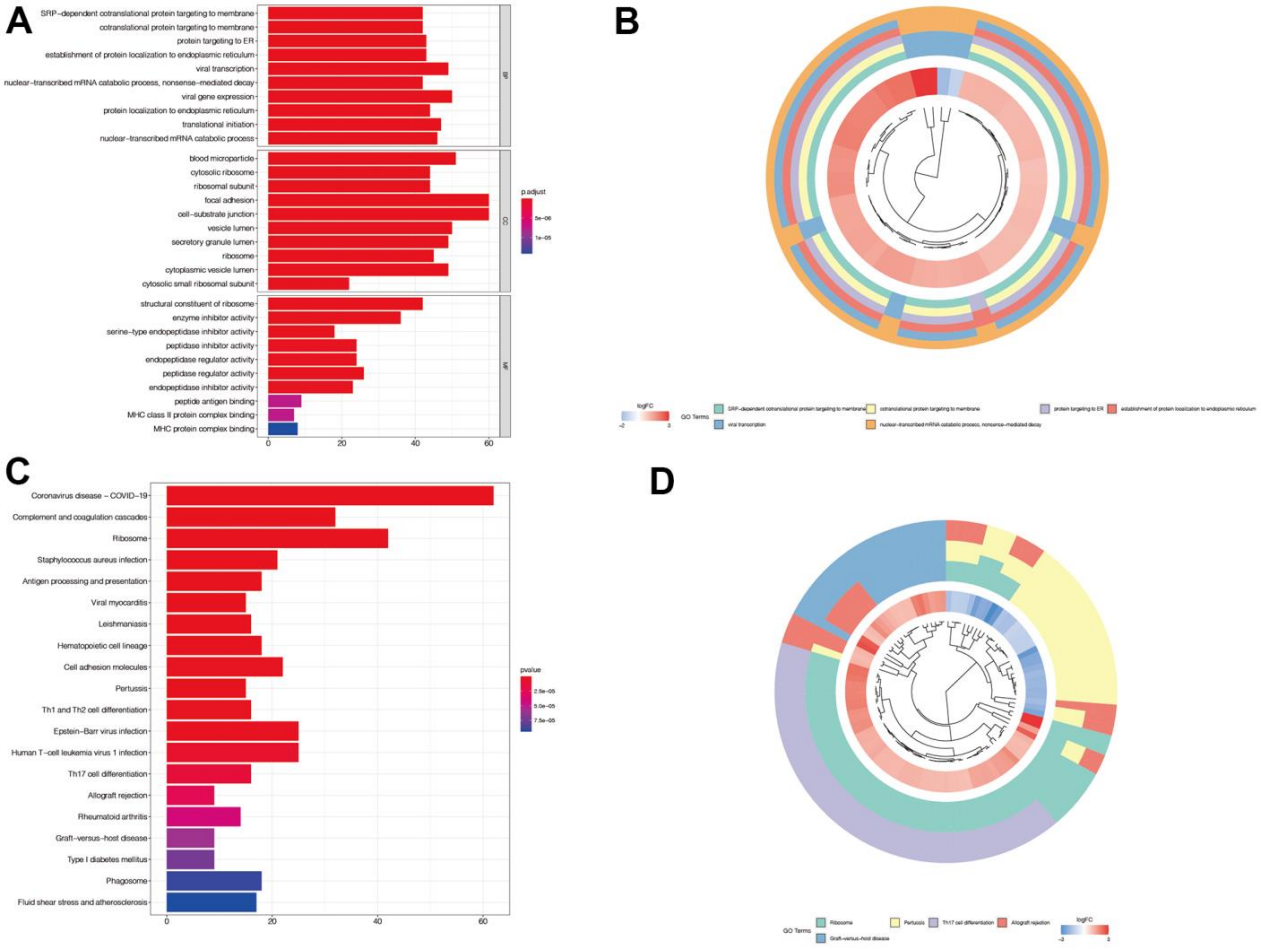
**Enrichment analysis of immune subtypes-related genes.** (**A**, **B**) In GO enrichment analysis, we found that these genes were mainly associated with protein translation and mRNA degradation. (**C**, **D**) In KEGG enrichment analysis, we found that these genes were mainly associated with antigen presentation and immune cell differentiation.

We then performed enrichment analysis of the genes associated with immune subtypes, and presented the first five important gene sets as a circle diagram. We found that these genes were mainly associated with protein translation and mRNA degradation (Figure 3A, 3B). Figure 3C, 3D illustrates that these genes were mainly associated with antigen presentation and immune cell differentiation.

### Construction of the prognostic model

**Figure 4:**
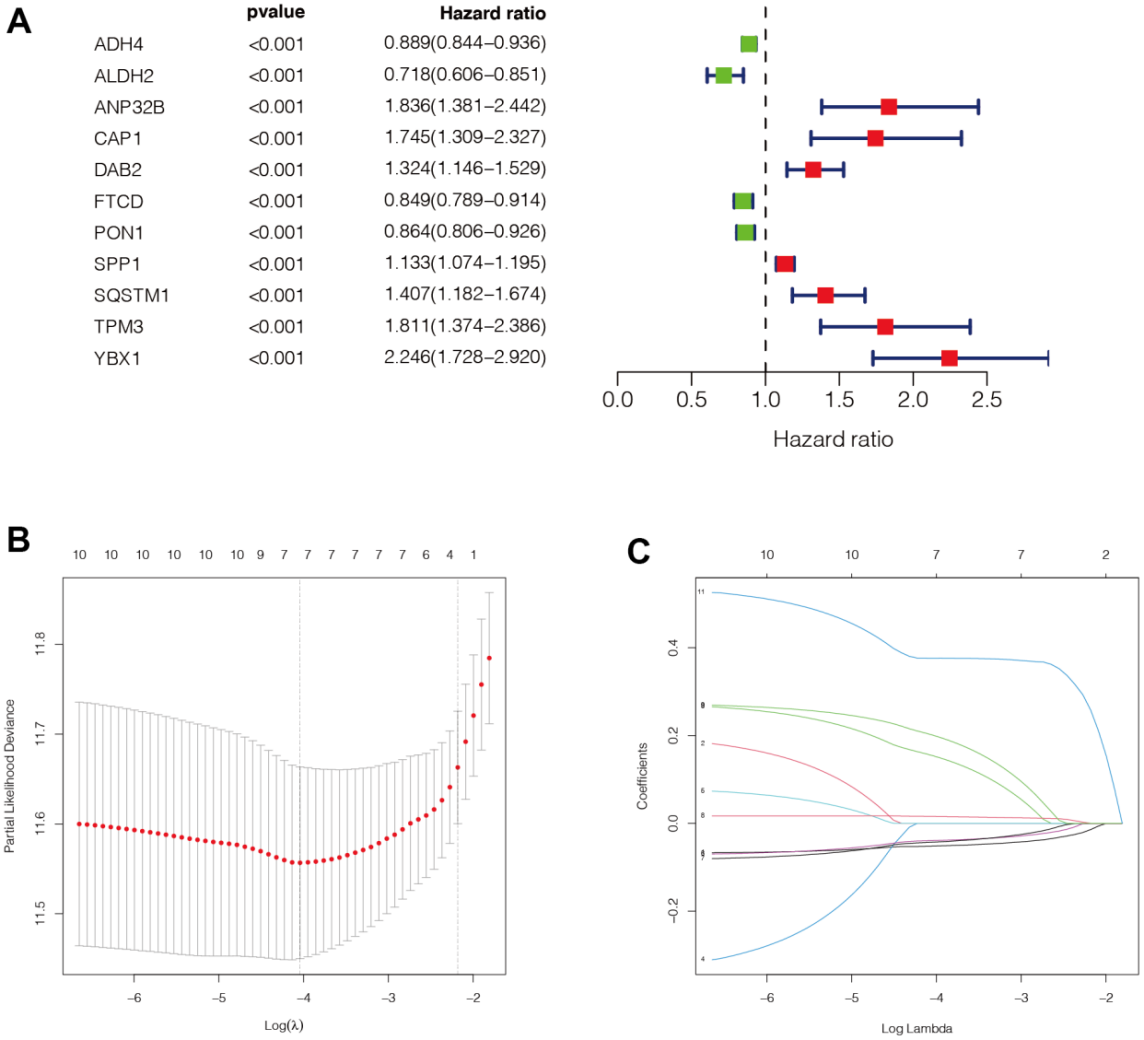
**Construction of the prognostic model.** (**A**) Through univariate COX analysis, we finally obtained 11 genes related to immune subtypes with prognostic values. There were 7 genes with HR>1, which were marked in red, and 4 genes with HR<1, which were marked in green. (**B**, **C**) Then, we performed LASSO regression analysis on these 11 genes, and the minimum lambda value was 0.023. We finally obtained 7 modeling genes, which were ADH4, ANP32B, FTCD, PON1, SPP1, SQSTM1, and YBX1. Risk score = ADH4*(-0.050) + ANP32B*0.136 + FTCD*(-0.038) + PON1(-0.040) + SPP1*0.015 + SQSTM1*0.182 +YBX1*0.376. We then divided the sample into high-risk groups and low-risk values based on the median risk score.

Through univariate Cox analysis, we finally obtained eleven genes related to immune subtypes with prognostic values. As shown in Figure 4A, there were seven genes with hazard ratio (HR) >1, which were marked in red, and four genes with HR <1, which were marked in green. Then, we performed Lasso regression analysis on these eleven genes, and the minimum lambda value was 0.023. We finally obtained seven modelling genes (Figure 4B, 4C), which were ADH4, ANP32B, FTCD, PON1, SPP1, SQSTM1, and YBX1. Risk score = ADH4*(-0.050) + ANP32B*0.136 + FTCD*(-0.038) + PON1(-0.040) + SPP1*0.015 + SQSTM1*0.182 +YBX1*0.376. We then divided the sample into high-risk and low-risk groups, based on the median risk score.

### Evaluation of the value of the prognostic model

**Figure 5:**
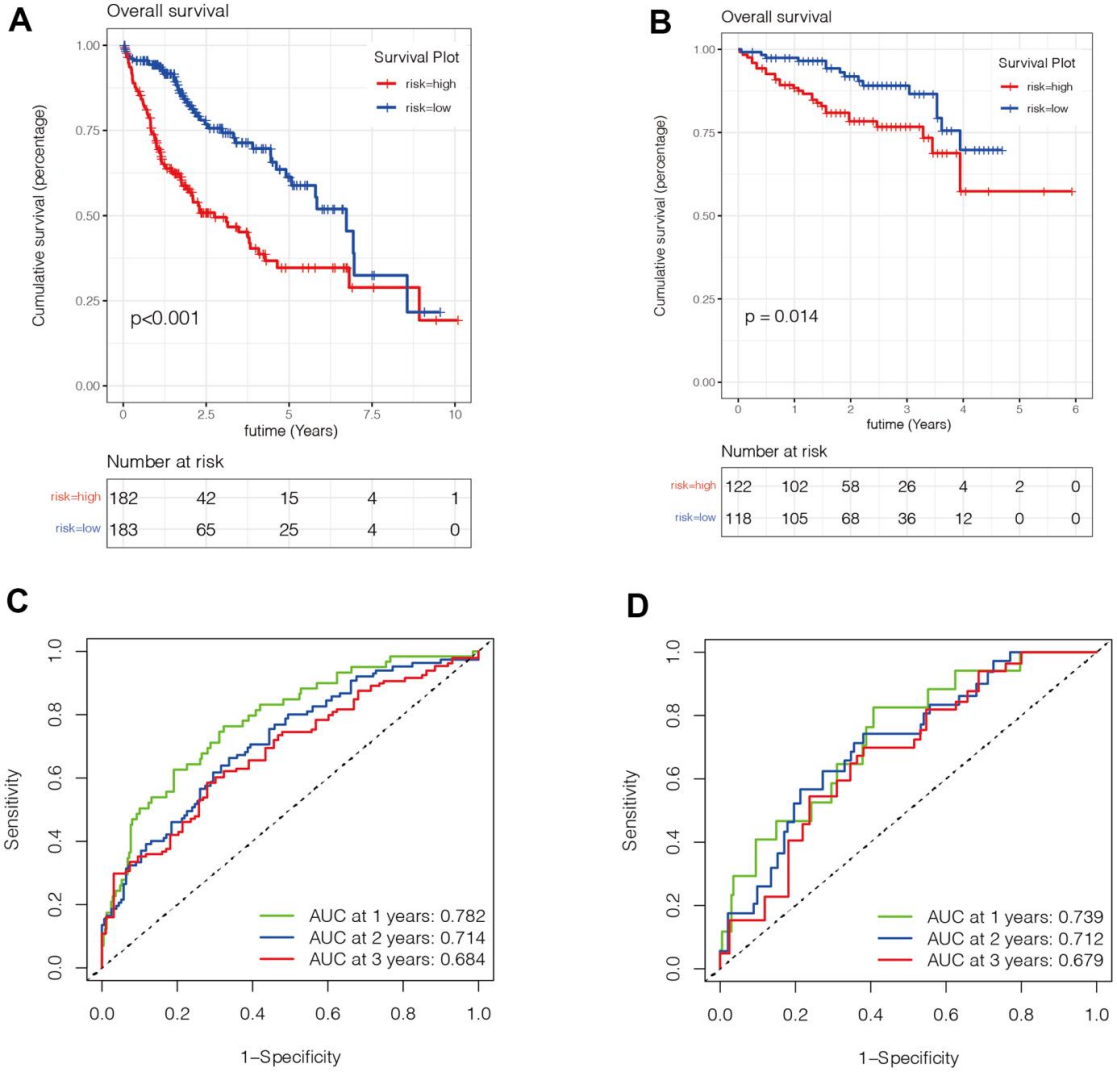
**Evaluation of the value of the prognostic model.** (**A**, **B**) We found that patients in the high-risk group had a poorer prognosis in both the TCGA (Figure 5A) and ICGC (Figure 5B) datasets (P <0.05). (**C**, **D**) To verify the accuracy of the risk score in prognostic diagnosis, we plotted the ROC curves for 1, 2, and 3 years in two data sets. We found that the area under the ROC curve of 1 year and 2 years in both data sets was greater than 0.7, and the area under the ROC curve of 3 years was close to 0.7, suggesting that the prognostic model has good stability and accuracy in predicting the prognosis of patients.

**Figure 6:**
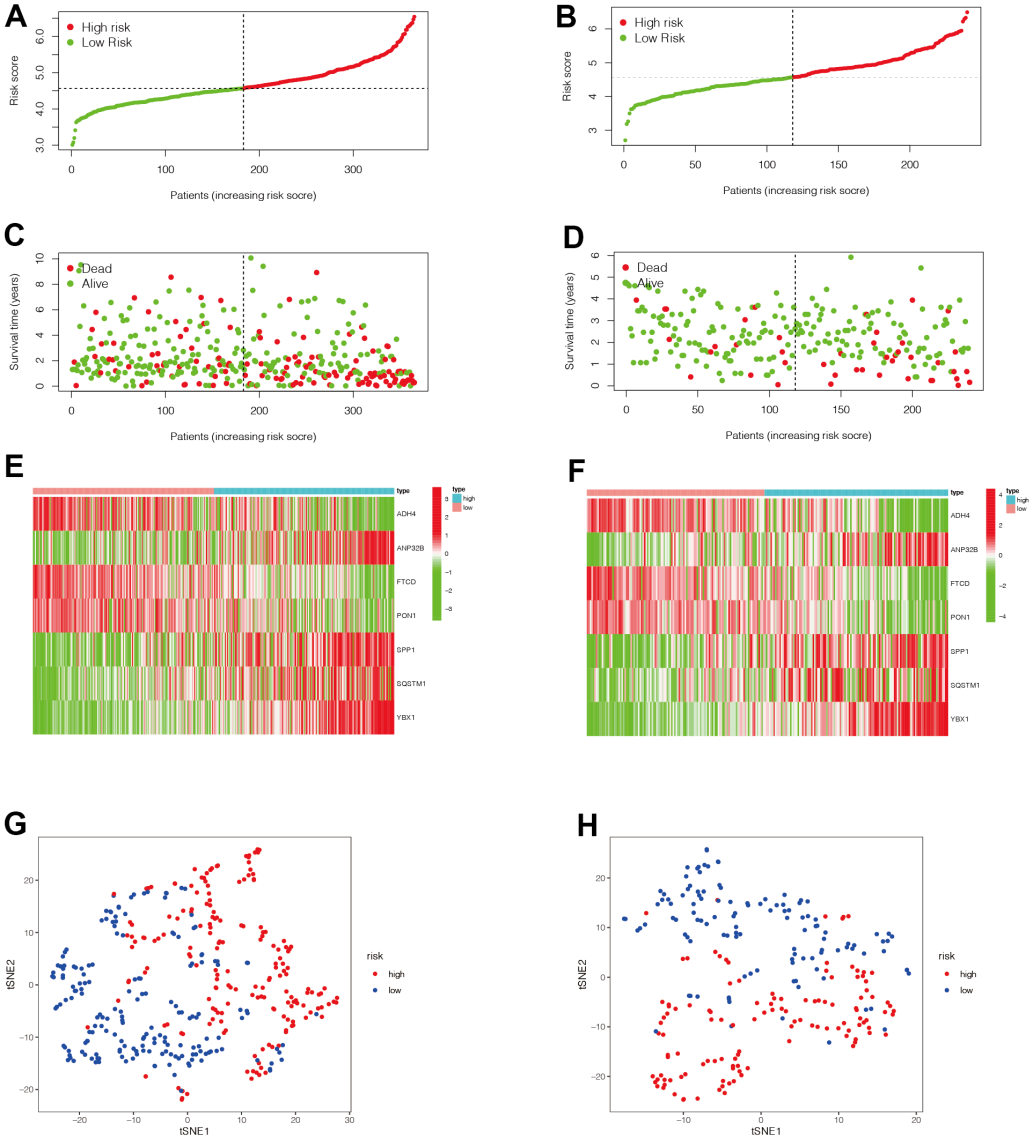
**Evaluation of the value of the prognostic model.** (**A**–**F**) We then analyzed the distribution of gene expression and patient survival in the models between the high - and low-risk groups in two data sets. We found that genes ADH4, FTCD, and PON1 were highly expressed in the low-risk group, while ANP32B, SPP1, SQSTM1, and YBX1 were highly expressed in the high-risk group. Moreover, we found that with the increase of risk value, the proportion of HCC patients who died increased. (**G**, **H**) We then further explored the ability of the prognostic model to distinguish HCC patients in two data sets and found that HCC patients could be well divided into two categories in both data sets.

We then evaluated the value of the model. In Figure 5A, 5B, we found that patients in the high-risk group had a poorer prognosis in both the TCGA and ICGC datasets (P <0.05). Then, to verify the accuracy of the risk score in prognostic diagnosis, we plotted the ROC curves for 1, 2, and 3 years of the two data sets (Figure 5C, 5D). We found that the area under the curve (AUC) for 1 year and 2 years in both data sets was greater than 0.7, and the AUC for 3 years was close to 0.7, suggesting that the prognostic model has good stability and accuracy in predicting the prognosis of patients. We found that genes ADH4, FTCD, and PON1 were highly expressed in the low-risk group, while ANP32B, SPP1, SQSTM1, and YBX1 were highly expressed in the high-risk group (Figure 6A–6F). Moreover, with the increase of risk value, the proportion of HCC patients who died increased. We then further explored the ability of the prognostic model to distinguish HCC patients in two data sets, and found that HCC patients could be divided into two categories in both data sets (Figure 6G, 6H).

### Analysis of immune infiltration and immune checkpoint

**Figure 7:**
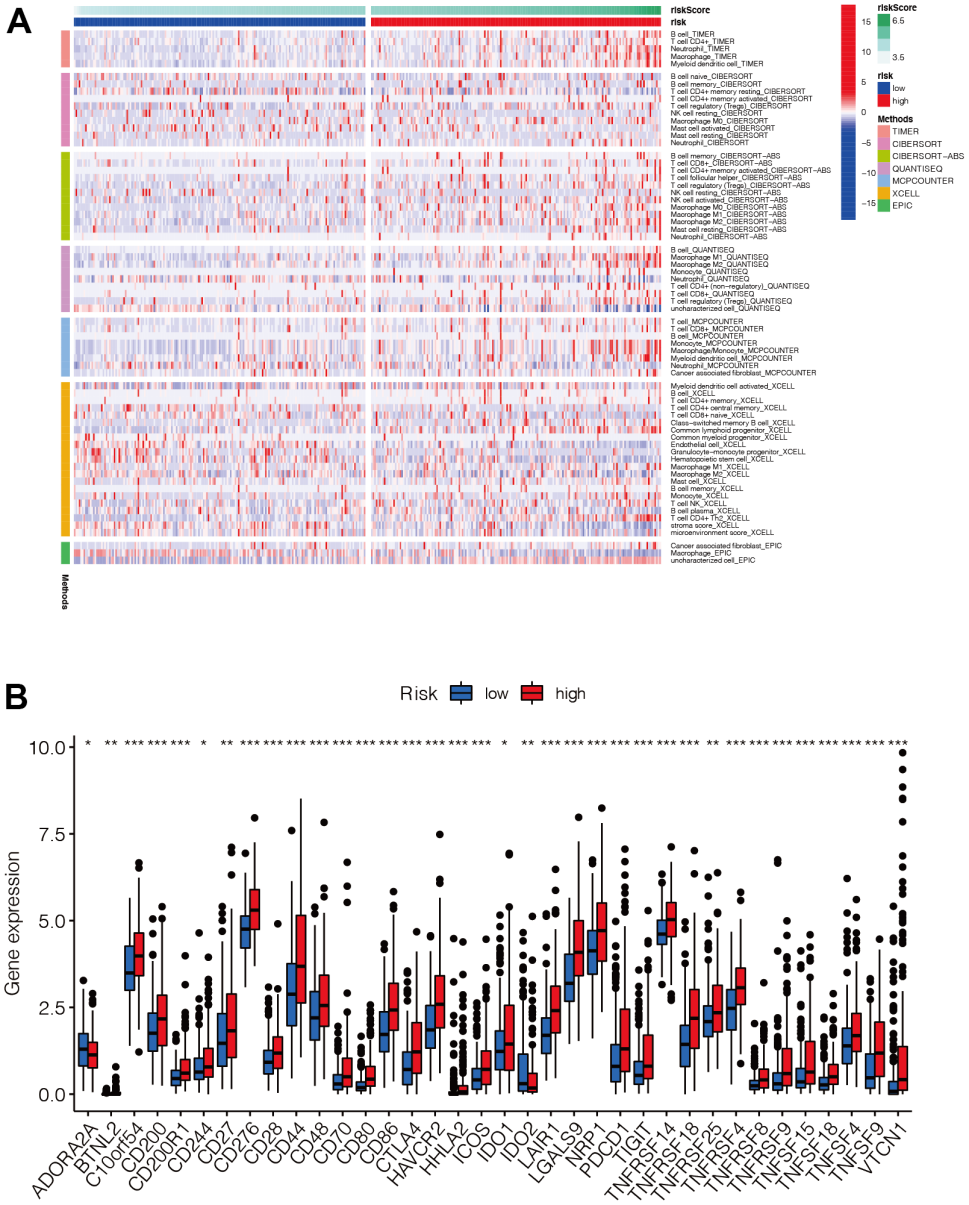
**Analysis of immune infiltration and immune checkpoint.** (**A**) The distribution of immune cells was significantly different between the high- and low-risk groups. In general, T cells and B cells tended to be highly expressed mainly in the high-risk group. (**B**) Most of the immune checkpoint genes were up-regulated in the high-risk group, suggesting that there may be differences in the immune microenvironment between the high- and low-risk groups.

The distribution of immune cell infiltration was significantly different between the high- and low-risk groups (Figure 7A). In general, T cells and B cells tended to be highly expressed mainly in the high-risk group. In addition, most of the immune checkpoint genes were up-regulated in the high-risk group (Figure 7B), suggesting that there may be differences in the immune microenvironment between the high- and low-risk groups.

### Analysis of gene mutations in high- and low-risk groups

**Figure 8:**
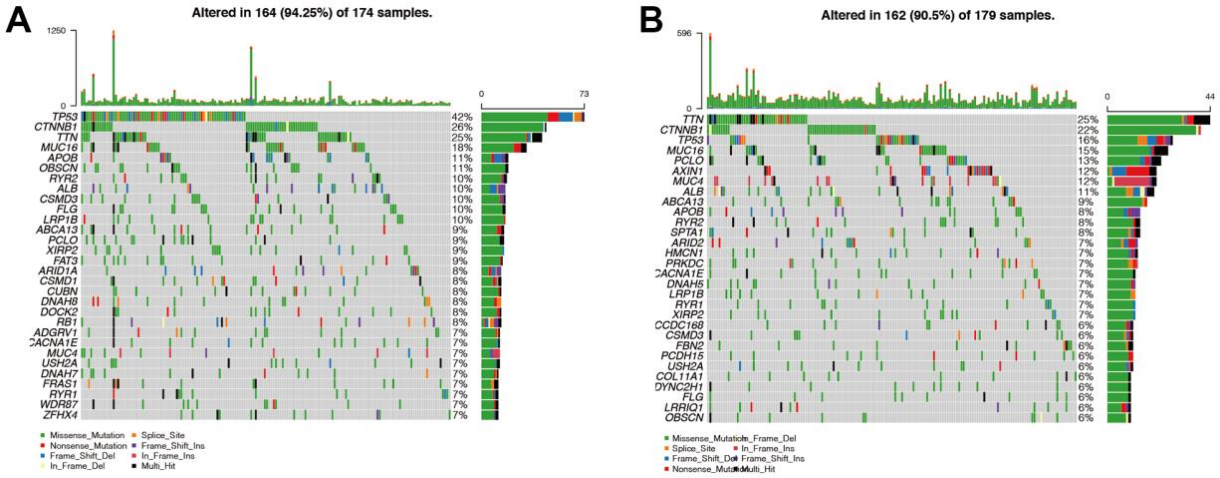
**Analysis of gene mutations in high- and low-risk groups.** (**A**, **B**) We found that the probability of mutation was 94.25% in the high-risk group (Figure 8A) and 90.5% in the low-risk group (Figure 8B). This suggested that patients in the high-risk group have more frequent mutations, possibly contributing to a poorer prognosis. TP53 was the most mutated gene in the high-risk group, and TTN was the most mutated gene in the low-risk group. Then we found that missense mutation was the main mutation type in both the high-risk group and the low-risk group. Interestingly, we found that the mutation types of AXIN1 and MUC4 in the low-risk group were mainly nonsense mutation and frame mutation.

The probability of gene mutation was 94.25% in the high-risk group and 90.5% in the low-risk group (Figure 8A, 8B). This suggested that patients in the high-risk group have more frequent mutations, possibly contributing to a poorer prognosis. TP53 was the most mutated gene in the high-risk group, and TTN was the most mutated gene in the low-risk group. The missense mutation was the main mutation type in both the high- and the low-risk groups. Interestingly, we found that the mutation types of AXIN1 and MUC4 in the low-risk group were mainly nonsense mutation and frame mutation.

### Pseudo-time series analysis

**Figure 9:**
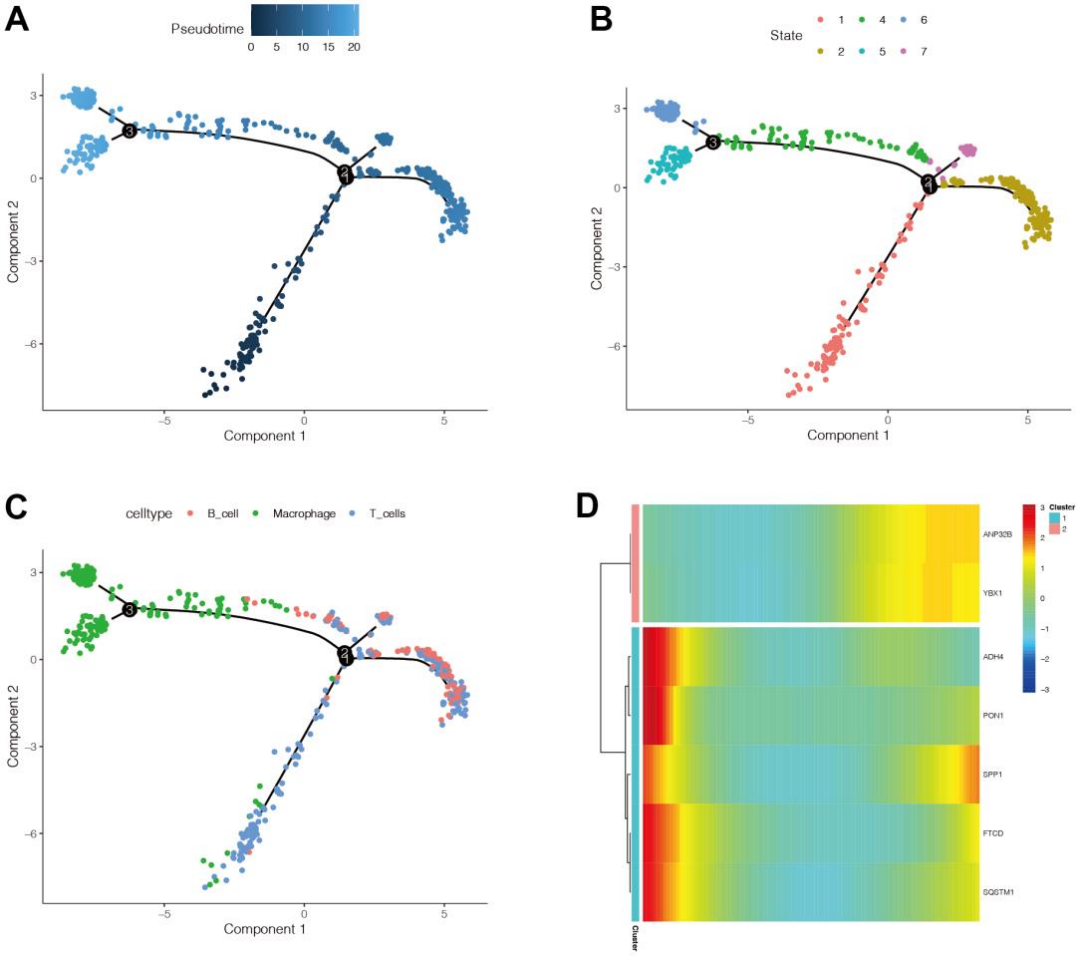
**The expression changes of modeling genes in immune cell differentiation.** (**A**) The darker the blue, the earlier the differentiation, and the lighter the blue, the later the differentiation. (**B**) There are six different differentiation states, and differentiation state 1 is the earliest. (**C**) With the differentiation of immune cells, there was a trend that T cells first differentiated to B cells, and then to macrophages. (**D**) It was found that the expression of ANP32B and YBX1 genes was mainly increased, the expression of SPP1 was mainly decreased and then increased, and the expression of ADH4, PON1, FTCD, and SQSTM1 was mainly down-regulated.

Pseudo-time series analysis on all HCC immune cells revealed that there are six different differentiation states, and differentiation state 1 is the earliest (Figure 9B). T cells first differentiated to B cells (Figure 9A), and then to macrophages (Figure 9C). The darker the blue, the earlier the differentiation, and the lighter the blue, the later the differentiation. The expression of ANP32B and YBX1 genes was mainly increased, the expression of SPP1 was first decreased and then increased, and the expression of ADH4, PON1, FTCD, and SQSTM1 was mainly down-regulated (Figure 9D).

### Drug sensitivity analysis

**Figure 10:**
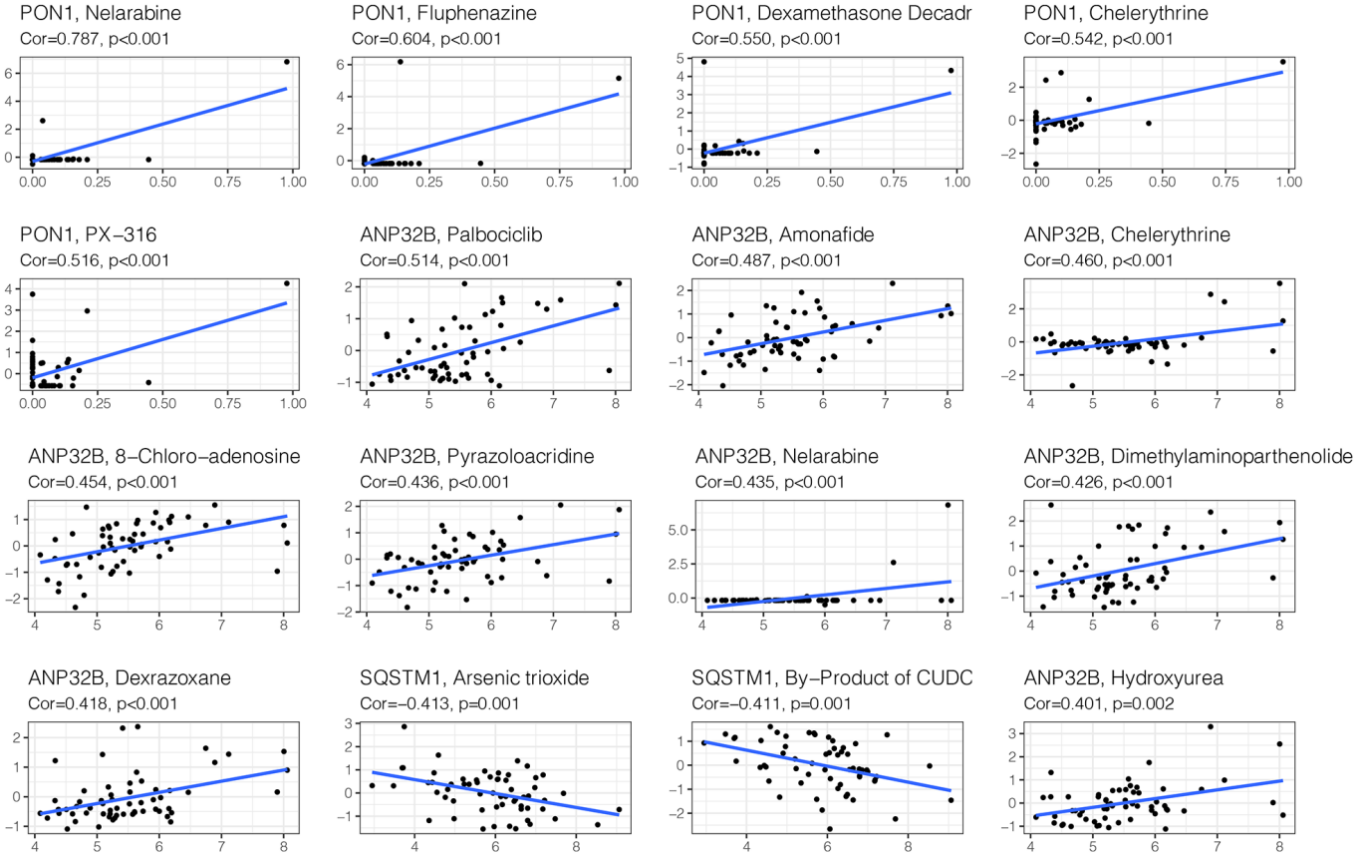
**Drug sensitivity analysis.** To improve the prognosis of HCC patients, we screened candidate drugs related to the prognosis model through the cellMiner website. The candidates are Nelarabine, Fluphenazine, Dexamethasone Decadr, etc.

To improve the prognosis of HCC patients, we screened candidate drugs related to the prognosis model through the cellMiner website. As shown in Figure 10, the candidates were nelarabine, fluphenazine, dexamethasone, and decadr.

### Construction of the nomogram

**Figure 11:**
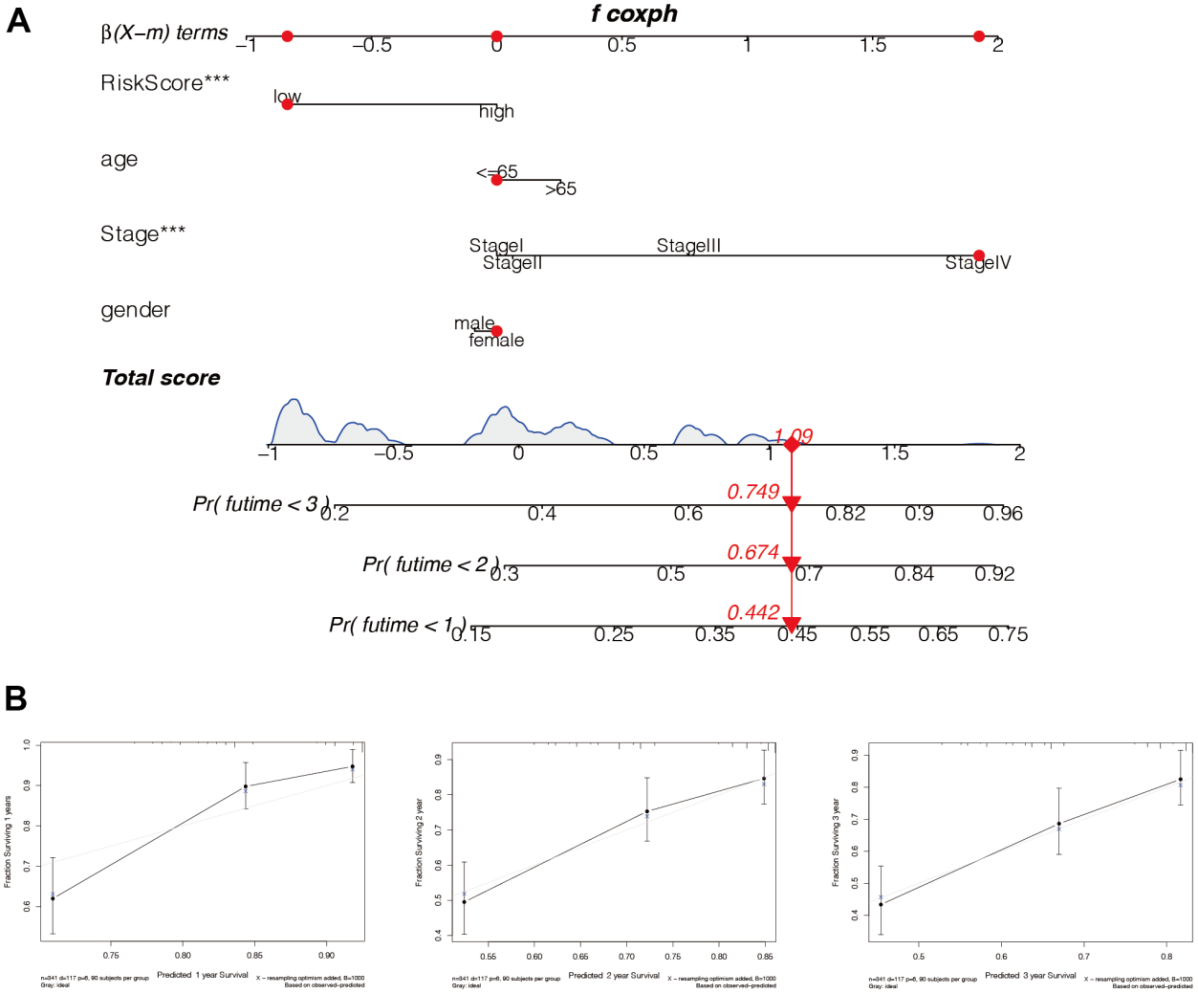
**Construction of the nomogram.** (**A**) The model risk score, age, tumor stage, gender, and other factors were comprehensively analyzed to construct the Nomo diagram. Combined with the clinical data and risk score of the patient “TCGA-RC-A6M5”, we predicted 1-year, 2-year, and 3-year mortality rates of 0.442, 0.674, and 0.749, respectively. (**B**) In addition, we constructed the calibration curves and found that this nomogram could predict the prognosis of HCC patients at 1, 2, and 3 years with good accuracy.

To further predict the prognosis of HCC patients, parameters including age, tumour stage, gender, and other factors were comprehensively analysed to construct the Nomo diagram (Figure 11A). Combined with the clinical data and risk score of the patient “TCGA-RC-A6M5”, we predicted 1-year, 2-year, and 3-year mortality rates at 0.442, 0.674, and 0.749, respectively. In addition, we constructed calibration curves (Figure 11B) and found that this nomogram could predict the prognosis of HCC patients at 1, 2, and 3 years with good accuracy.

### Quantitative real-time polymerase chain reaction (qRT-PCR)

**Figure 12:**
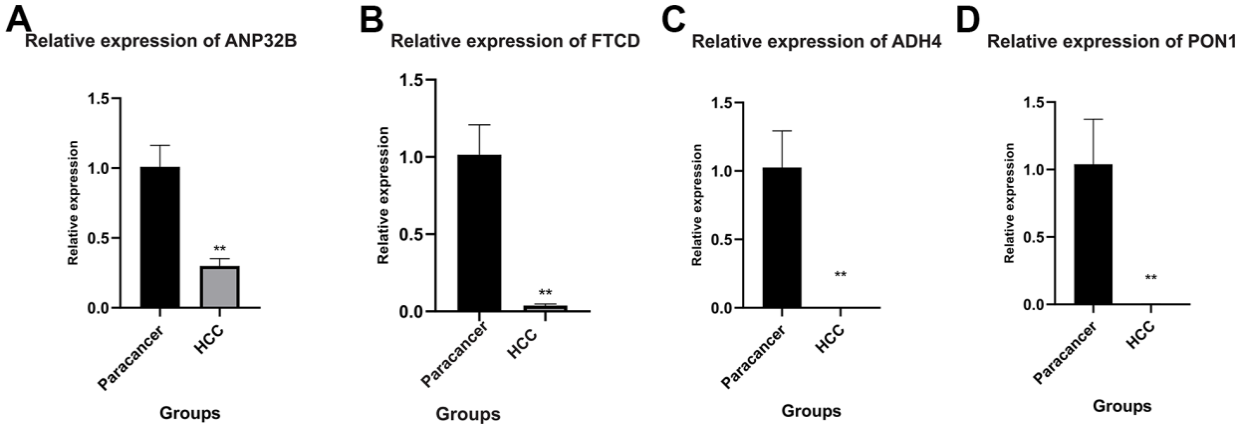
**Quantitative real-time polymerase chain reaction (qRT-PCR).** (**A**–**D**) ANP32B, FTCD, ADH4 and PON1 were differentially expressed in HCC cells and normal para-cancer tissues. The expression levels of these four genes were all downregulated in HCC, and the expression levels of ADH4 and PON1 were very low. (**P<0.01).

As the expression analysis of the 7 model genes mentioned above was only performed in the high-risk and low-risk groups of HCC patients. Next, PCR assay was used to detect the expression of 7 model genes in HCC cells and normal adjacent tissues. As shown in Figure 12, among the 7 model genes, 4 genes were differentially expressed in HCC cells and normal para-cancer tissues, namely, ANP32B, FTCD, ADH4 and PON1 (Figure 12A–12D). The expression levels of these four genes were all downregulated in HCC, and the expression levels of ADH4 and PON1 were very low.

## DISCUSSION

The development of HCC is closely related to the immune system [13]. On the one hand, liver diseases involving immune disorders, such as viral hepatitis and non-alcoholic fatty liver, play an important role in the pathogenesis of HCC [14]. The liver is an immune organ that is exposed to many of its own and foreign antigens [15]. Thus, the immune microenvironment of HCC is large and complex [16]. Currently, immunotherapy is gaining momentum in HCC, but there is still a significant proportion of patients with low reactivity [17]. This suggests that our understanding of the immune microenvironment of hepatocellular carcinoma is far from sufficient. It is of profound significance to explore the complex immune microenvironment of hepatocellular carcinoma, identify novel biomarkers, and construct a meaningful prognostic model.

In this present study, we first performed reduction and cluster analysis on single-cell sequencing data. HCC was divided into immune and non-immune subtypes. We then identified differentially expressed genes in the two subpopulations. Cox and Lasso regressions were performed on the differentially expressed genes to construct a prognostic model based on immune subtypes. The model divided HCC patients into high-risk and low-risk groups through the calculation of risk scores. Patients in the high-risk group had a significantly poorer prognosis. The prognostic guiding significance was verified in the validation set, and the higher AUC value indicated higher accuracy. Subsequently, we identified that the differences in the tumour microenvironment between the high-risk and the low-risk groups related to immune infiltration, signal activation, drug sensitivity, etc. Finally, PCR was used to explore the expression of model genes in HCC and normal tissues.

Despite the initial success of immunotherapy in HCC, unfortunately only a fraction of patients benefit from immunotherapy, due to the apparent heterogeneity of HCC [18]. Genomic instability, disruption of molecular and signal transduction networks, microenvironmental differences, and the presence of tumour stem cells are all sources of HCC heterogeneity [19]. In this context, individualized precision treatment is particularly important. Advanced sequencing techniques, especially current single-cell sequencing techniques, provide a way to better understand the heterogeneity of HCC [20]. In our study, HCC cells were divided into immune and non-immune groups by single-cell sequencing analysis, and the differences in gene expression between the two groups were explored. This is undoubtedly beneficial to the understanding of the heterogeneity of HCC.

As mentioned earlier, HCC is a tumour type with a poor prognosis. Therefore, some emerging prognostic markers can be identified to improve the prognosis of HCC patients. We present a new prognostic model based on immune typing. For patients in the high-risk group, more aggressive treatment should be adopted to improve patient outcomes. We also studied the differences in immune cell infiltration between the two groups, from which we can provide references to guide immunotherapy of HCC.

Accurate identification of HCC patients likely to respond to immunotherapy is critical [21]. Tumour mutation load (TMB), an indicator of immunogenicity in patients with HCC, is an emerging prognostic and therapeutic response marker [22, 23]. By measuring TMB, we were able to identify the ability of patients with HCC to benefit from immunotherapy. In our study, we explored the difference in TMB between the high-risk and low-risk groups in this prognostic model, which has implications for our further targeted treatment of HCC.

In summary, our study establishes a novel and effective immune subtype-related prognostic model for HCC. These results have implications for the precise treatment of HCC and the exploration of the tumour immune microenvironment. However, our research has limitations. We lack relevant functional tests to further verify the function of model genes, which we will improve in the future.

## CONCLUSIONS

We constructed a prognostic model based on immune subtypes in HCC. This model can accurately assess the prognosis of HCC patients to guide their treatment. Moreover, our study can provide new ideas for immunotherapy directed at HCC.

## MATERIALS AND METHODS

### The data source

Transcriptome data from 424 HCC samples and clinical data from 377 HCC patients from The Cancer Genome Atlas Program (TCGA) were downloaded. Transcriptomic data types were respectively LIHC-count and LIHC-FPKM, where the Count format could be used for subsequent difference analysis, and FPKM format could be used for subsequent analysis after conversion to TPM. We also downloaded the mutation data for the HCC patients, with the data type of MuTect2 Variant Aggregation and Masking. In addition, we have downloaded transcriptome and clinical data from The Cancer Genome Collaboratory (ICGC) website. The Gene Expression Omnibus (GEO) database (https://www.ncbi.nlm.nih.gov/geo/) were used to download the single-cell sequencing dataset GSE146115 of HCC.

### Transcriptome data processing

Firstly, we matched the HCC samples of TCGA with the clinical data, excluded the subjects with 0 survival time, and finally obtained 365 patients with both transcriptome and clinical data. Then, 240 HCC patients with both transcriptome and clinical data were obtained, by matching the transcriptome data obtained from the ICGC database with clinical data, and excluding normal samples and HCC subjects with 0 survival time. Finally, we used the “Combat” function of the R package “SVA” to conduct the debatch-effect of the two sequencing data sets, and took the intersection of the genes in the two data sets. Finally, the expression matrix of 12,757 genes in 365 HCC patients from the TCGA dataset was obtained, and the expression matrix of 12,757 genes in 240 HCC patients from the ICGC dataset was obtained.

### Quality control of single-cell sequencing data

The following conditions were set: the number of genes in each cell is in the range of 300 to 7000, the percentage of mitochondria is <10%, the percentage of red blood cells is <3%, and the total gene expression copy numbers are less than 300,000. We selected the most highly variable 3,000 genes and labelled them in red, and we tagged the names of the first ten genes that were highly variable at the same time.

### Dimension reduction and cluster analysis

Firstly, principal component analysis (PCA) dimension reduction was carried out based on the highly variable genes, and the number of PCs was set to six, and then a total of eight clusters were obtained. These clusters were presented in uMAP format, and the first ten genes with significant differences in each cluster were selected and mapped. We split these clusters into immune subgroups and non-immune subgroups based on the immune cell marker genes “COL2A1”, “PTRPC”, and “EPCAM”. After obtaining function genes related to immune subgroup by FindMarkers, we set the filter to | avg_log2FC | > 2 and p_val_adj < 0.05.

### Gene function enrichment and pathway enrichment analysis

The characteristic genes obtained that related to immune subsets were subjected to enrichment analysis by GOplot package. An enrichment result of P <0.05 was retained, and the first five important gene sets were displayed in the form of a circle diagram.

### Pseudo-time series analysis

Pseudo-time series analysis were performed to observe the sequence of cell differentiation through the “Monocle” package. We used the sample function to randomly select 1,500 cells for subsequent analysis. The DDRTree method was used to perform dimension reduction analysis. Finally, the plot_cell_trajectory function was used to display the results.

### Construction of a prognostic model

In the transcriptomic data of TCGA, we matched the obtained characteristic genes related to immune subtypes to obtain the corresponding gene expression. We then performed univariate Cox analysis (the screening condition was set at P <0.0002) to identify prognostic genes associated with immune subsets, and displayed them in the form of forest maps. Then, we performed Lasso regression analysis on these genes, set the parameter of the family as “Cox” and the number of MaxIT as 1000, and used the obtained characteristic genes for model construction. At the same time, we calculated the risk score of the TCGA dataset according to the formula of the model, and stratified it into high-risk group and low-risk groups via the median score for subsequent analysis.

### Evaluation of the model

First the prognostic effect of this model was evaluated by plotting the survival curves of high- and low-risk groups in the TCGA and the ICGC datasets. Since the prognosis of liver cancer is generally poor, it may be more meaningful to plot the receiver operating characteristic (ROC) curve within three years. Therefore, the timeROC package were used to draw time-dependent ROC curves for 1, 2, and 3 years, to observe the accuracy and stability of our model. We then used heat maps to compare the expression of the model genes between the high- and low-risk groups, and plotted the risk curve. We plotted the distribution of patients between high and low-risk groups using the Rtsen package, to determine the effectiveness of differentiating patients by risk value.

### Analysis of immune infiltration level and immune checkpoint

First the analysis results of seven types of immune cell infiltrates in HCC patients were downloaded from the TCGA database in the Timer 2.0 database. By matching HCC samples, we presented the results in the form of heat maps of immune cells that were expressed differently (P <0.05) between the high- and low-risk groups. At the same time, we also obtained a group of immune checkpoint-related genes from the literature, and then showed the immune checkpoint genes with different expressions between the high- and low-risk groups (P <0.05) in the form of a bar chart.

### Analysis of gene mutation between high- and low-risk groups

First patients in the high- and low-risk groups were selected and the MafTools package were used to show the mutations in the first 30 genes in a waterfall plot pattern, to see the mutations between the high- and low-risk groups.

### Pseudo-time series analysis

We used the Monocle package again to perform pseudo-time series analysis on all immune cells using the DDRTree method, and the plot was displayed using the plot_cell_trajectory function. At the same time, the plot_preudotime_heatmap function was used to display the expression changes of modelled genes, along with the differentiation of immune cells.

### Drug sensitivity analysis

The drug sensitivity data of HCC was downloaded from the cellMiner database, and the drugs related to the model genes were obtained by Pearson correlation analysis. The drugs were ranked in order of the absolute value of correlation, and the first 16 with strong correlation were displayed.

### The construction of the nomogram

We used the Regplot package to include clinical characteristics and risk groups, and build regression models. Clinical data and risk score markers for patients with “TCGA-RC-A6M5” were used to predict mortality at 1, 2, and 3 years. Then, to assess the accuracy of the predictions, we used the “RMS” package for analysis, and plotted the calibration curves for 1, 2, and 3 years.

### Quantitative real-time polymerase chain reaction (qRT-PCR)

As the expression analysis of the model genes mentioned above was only performed in the high-risk and low-risk groups of HCC patients. Next, PCR assay was used to detect the expression of model genes in HCC cells and normal adjacent tissues. Total cellular RNAs were isolated from cells using Trizol Reagent (Invitrogen, Carlsbad, CA, USA) according to the manufacturer’s instructions. The reverse transcription was conducted using the reverse transcription kit provided by Takara (Otsu, Shiga, Japan). Real-time polymerase chain reaction (RT-PCR) was performed using a QuantiTect SYBR Green PCR Kit (Takara), and on a Applied Biosystems QuantStudio 1 (Thermo, Waltham, MA, USA). Relative quantification was determined using the -2^ΔΔCt^ method. The relative expression of messenger RNA (mRNA) for each gene was normalized to the level of glyceraldehyde-3-phosphate dehydrogenase (GAPDH) mRNA. Primer sequences were summarized in Supplementary Table 1.

### Compliance with ethical standards

All procedures performed were in accordance with the declaration of the ethical standards of the institutional research committee and with the 1964 Helsinki 387 Declaration and its later amendments. The ethics committee has approved this study of the First Affiliated Hospital of Nanjing Medical University.

### Data availability statement

The data that support the findings of this study are available in GEO database at [https://www.ncbi.nlm.nih.gov/geo/], reference number [GSE146115].

## Supplementary Material

Supplementary Table 1
