# Supplementary material for: An immune subtype-related prognostic signature of hepatocellular carcinoma based on single-cell sequencing analysis
Source: Aging (Albany NY). 2022 Apr 12;14(7):3276–92. doi: 10.18632/aging.204012 (PMC9037256; doi:10.18632/aging.204012)
Supplement: Supplementary Table 1 [file aging-14-204012-s001.pdf]

SUPPLEMENTARY TABLE

Supplementary Table 1. Primer sequences.

Mus Mucus GAPDH_F	AGGTCGGTGTGAACGGATTTG
Mus Mucus GAPDH_R	TGTAGACCATGTAGTTGAGGTCA
ADH4-F	AGTTCGCATTCAGATCATTGCT
ADH4-R	CTGGCCCAATACTTTCCACAA
ANP32B-F	CTGTTCGAGAACTTGTCTTGGAC
ANP32B-R	AGCTTGGGGAGATTTGAAACTG
FTCD-F	GGAATGCGTCCCCAACTTTTC
FTCD-R	TGTCGATAAGTCGGGAAGCTAC
PON1-F	TCCGAGAGGTACAACCCGTAG
PON1-R	CCAGTCCATTAGGCAGTATCTCC
SPP1-F	CTCCATTGACTCGAACGACTC
SPP1-R	CAGGTCTGCGAAACTTCTTAGAT
SQSTM1-F	GACTACGACTTGTGTAGCGTC
SQSTM1-R	AGTGTCCGTGTTTCACCTTCC
YBX1-F	GGGGACAAGAAGGTCATCGC
YBX1-R	CGAAGGTACTTCCTGGGGTTA
